# Absence of Both Right and Left Main Coronary in a COVID Survivor

**DOI:** 10.3390/diagnostics11071199

**Published:** 2021-07-01

**Authors:** Marian Pop, Krisztina Pal, Diana Vaga

**Affiliations:** 1 Radiology Department, Tirgu Mures Emergency Institute for Cardiovascular Diseases and Heart Transplant, 540136 Tirgu Mures, Romania; 2 ME1 Department, “George Emil Palade”, University of Medicine, Pharmacy, Sciences and Technology of Tirgu Mures, 540142 Tirgu Mures, Romania; 3 Cardiology Department, Tirgu Mures Emergency Institute for Cardiovascular Diseases and Heart Transplant, 540136 Tirgu Mures, Romania; dr.krisztinapal@gmail.com; 4 Radiology Department, Tirgu Mures Emergency Clinical County Hospital, 540136 Tirgu Mures, Romania; vaga_diana@yahoo.com

**Keywords:** coronary CT angiography, coronary anomaly, absent right coronary artery, congenital cardiovascular

## Abstract

The prevalence of isolated right coronary artery (RCA) absence ranges from 0.014% to 0.066% in the general population, but its combination with an absent left main (dual ostium left anterior descending [LAD] and super-dominant left circumflex [LCx]) has not been previously described. We report the case of a rare coronary artery anomaly: an absent RCA with LAD and LCx coronary arteries arising separately from the left coronary sinus. A 53-year-old male with recent COVID-19 infection was referred to our service for coronary computed tomography angiography (CCTA) due to the recent onset of atypical chest pain. The RCA was absent, with no vessel leaving the right or non-coronary sinus. The LAD and LCx emerged from the left coronary sinus, with a “double-barrel” appearance. The LAD was unremarkable, with small, non-stenosed calcified plaque. The LCx had a 3 mm diameter, arching downward in the left atrioventricular groove, passing through the crux cordis, continuing into the right atrioventricular groove, and ending as a left acute artery and sinonodal artery. No significant stenosis was found on any of the vessels, ruling out atherosclerotic coronary disease.

**Figure 1:**
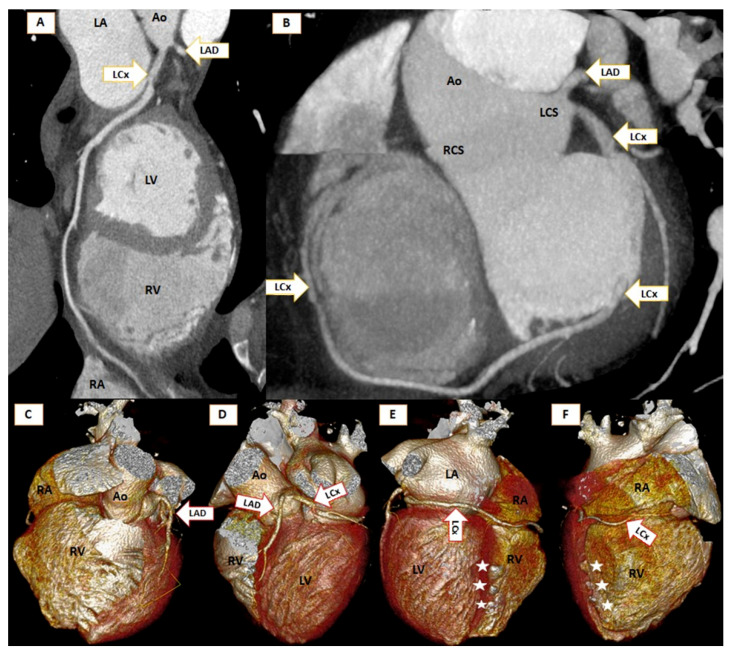
Panel (**A**): Curved multiplanar reconstruction images of the left circumflex artery (LCx) demonstrate its course around the atrioventricular groove. The left main coronary artery was also absent, with both left anterior descending (LAD) and LCx arising from the left coronary sinus with a “double-barrel” appearance. Regardless of the branches involved (right coronary artery, LAD, or LCx), the four classical coronary course variants are classified as normal, pre-pulmonary, interaorto-pulmonary, and retroaortic [1,2,3]. Panel (**B**): Maximum intensity projection of the left circumflex coronary artery (LCx) course. LCx emerges from the left coronary sinus and has a 3 mm diameter. It travels in the left atrioventricular (AV) groove, continues in the AV groove of the inferior aspect of the heart, and then ascends in the right AV groove to the right auricular ostium. Its ramifications include two marginal branches, a posterolateral branch, a thin posterior descending artery, and, from the right AV groove, an acute marginal branch (AM) and a branch to the sinoatrial node. A note is made of the missing right coronary artery (RCA). Panels (**C**–**F**): The 3D virtual rendering technique (VRT) illustrates the absence of the right coronary artery arising from the right coronary sinus, the absence of the left main coronary artery, and the double-barrel appearance. LCx travels around the heart in the AV groove. Panels (**E**,**F**) visualize three diverticular dilatations on the inferior side of the right ventricle (RV; white stars). Such diverticular dilatations of the RV are rare and have variable natural history, requiring supportive treatment unless they are causing symptoms. LA: Left atrium, LV: Left ventricle, RV: Right ventricle, RA: Right atrium, LCS: Left coronary sinus, RCS: Right coronary sinus, Ao: Aorta, LAD: Left anterior descending coronary artery, LCx: Left circumflex coronary artery, Five-pointed star: Right ventricle diverticulum.

## Data Availability

Data is available upon reasonable request.
